# Low temperature preservation of porcine semen: influence of short antimicrobial lipopeptides on sperm quality and bacterial load

**DOI:** 10.1038/s41598-020-70180-1

**Published:** 2020-08-06

**Authors:** B. Hensel, U. Jakop, K. Scheinpflug, K. Mühldorfer, F. Schröter, J. Schäfer, K. Greber, M. Jung, M. Schulze

**Affiliations:** 1 Institute for Reproduction of Farm Animals Schönow, Bernauer Allee 10, 16321 Bernau, Germany; 2 Leibniz Institute for Zoo and Wildlife Research, Alfred-Kowalke-Str. 17, 10315 Berlin, Germany; 3 Department of Cardiovascular Surgery, Heart Center Brandenburg, Brandenburg Medical School, Ladeburger Straße 17, 16321 Bernau bei Berlin, Germany; 4 Institute of Veterinary Medicine, University of Göttingen, Burckhardtweg 2, 37077 Göttingen, Germany; 5 Physical Chemistry Department, Faculty of Pharmacy, Medical University of Gdańsk, al. gen. J. Hallera 107, 80-416 Gdańsk, Poland

**Keywords:** Antimicrobials, Drug screening, Animal biotechnology, Cell biology

## Abstract

Antimicrobial resistance is a steadily increasing problem and poses a serious threat to global public health. Therefore, it is highly necessary to advance the development of novel antimicrobial compounds and semen preservation strategies. The aim of this study was to evaluate a low temperature, antibiotic-free preservation procedure using Androstar Premium (ASP) extender (Minitüb) with antimicrobial lipopeptides. Firstly, seven lipopeptides in two concentrations (1 × minimum inhibitory concentration (MIC)/2 × MIC) were tested on their sperm-compatibility at 17 °C. Two lipopeptides, C16-KKK-NH_2_ and C16-KKKK-NH_2_, did not negatively affect sperm quality and were further evaluated for their efficiency of bacterial growth inhibition at 5 °C. Besides an overall diminution of colony forming units, both peptides showed a reduction of bacterial subcultures (n = 103) with a decrement in Gram-positive rods from 65 (ASP w/o supplements) to 39/52 (ASP w/ C16-KKK-NH_2_/C16-KKKK-NH_2_), in Gram-positive cocci from 21 to 9/10 and in Gram-negative species from 17 to 8/5 total subcultures. Furthermore, lipopeptides revealed activity towards selected bacteria of potential concern in artificial insemination like *Trueperella pyogenes*, *Alcaligenes faecalis*, *Pseudomonas aeruginosa* (not C16-KKK-NH_2_), *Pasteurella* sp., *Providencia stuartii*, *Escherichia coli* (not C16-KKKK-NH_2_) and *Streptococcus porcinus* (not C16-KKKK-NH_2_). Consequently, both tested lipopeptides are promising candidates for alternative antibiotic-free preservation techniques of boar semen.

## Introduction

Spreading antimicrobial resistance is a growing problem in human and veterinary medicine, poses a serious threat to global public health^1^ and should therefore be addressed in a multifaceted approach from all areas of concern, including the industry branch of artificial insemination (AI) in pigs. It has been proven, that unhindered bacterial growth has detrimental effects on several sperm quality characteristics^2^ and a transmission of potential pathogens to the inseminated sow has to be prevented in order to minimize negative effects on fertility and litter size^3^. Semen collection is a non-sterile process and AI doses are conventionally stored at temperatures between 16 and 18 °C to prevent cold shock injuries to the highly sensitive boar spermatozoa^4^. The combination of moderate temperatures and the use of nutrient-rich semen extenders promotes bacterial growth of psychrophilic and mesophilic species^5^. As a countermeasure, antibiotics like gentamicin sulphate are routinely added to boar semen preserved for AI (Council Directive, European Union, 90/429/EEC).

A steadily growing portion of contaminant bacteria isolated from AI doses are resistant to antibiotics commonly used as additives in semen extenders^6^, threatening animal welfare. This has far-reaching consequences for public health because of the natural backflow of parts of the rather large-volumed AI doses after insemination^7^, causing antibiotics to reach the liquid manure. Therefore, it is highly necessary to question the standardized supplementation of boar semen extenders with conventional antibiotics^6^. In the search for strategies to prevent antibiotic resistance, antimicrobial peptides (AMPs) and their potential to serve as a substitute for conventional antibiotics have been the focus of many research projects over the past ten years^8–11^.

AMPs are a structurally diverse group of cationic, amphiphilic peptides. Bacteria are less likely to develop resistance towards AMPs than towards conventional antibiotics as the toxic pathways of AMPs often provoke cell lysis through unspecific disruption of the lipid bilayers rather than by an interaction with a specific target^12^. Ideally, AMPs used for boar semen preservation should possess the following criteria: (1) broad spectrum of antimicrobial action, (2) absence of sperm toxicity, (3) no interference with fertility, (4) high stability (for the entire storage duration of boar spermatozoa), (5) high activity at common semen storage temperatures, (6) low potential to evoke resistance, (7) ease of application and (8) economic feasibility^13^. Even though there have been promising results regarding the use of AMPs in AI in pigs, there are also many challenges, like the expensive and often time-consuming synthesis, insufficient antimicrobial activity or detrimental effects on sperm quality^14^. Another approach towards the waiving of antibiotics in the pig AI industry is the low temperature storage of extended boar spermatozoa, which helps to minimize bacterial growth^15^ and could also help to reduce the development of multi-resistant bacteria with absent selection pressure of antibiotics.

Thus, the aim of the present study was to evaluate a holistic approach combining a low temperature, antibiotic-free storing procedure for boar sperm with the use of alternative antimicrobial agents. The applied substances were a group of seven short lipopeptides derived from the two amino acid derivatives Fmoc-Lys(Boc)-OH and Fmoc-Lys(Fmoc)-OH^16,17^. Short lipopeptides possess many of the qualities of the more in-depth studied AMPs stated above (amphipathic, positively charged, antimicrobially active) while being less cost- and time-consuming in production^18^. All tested lipopeptides had a positive net charge of at least two and a supplemented fatty acid chain in order to create a proper balance between the hydrophilic and hydrophobic entities of the molecule, which is a crucial factor for their antimicrobial potency^17^. After testing the different lipopeptides on their influence on sperm quality, the combination of a cold-temperature storage at 5 °C with the addition of the most sperm-compatible lipopeptides was examined on synergistic effects concerning the inhibition of bacterial growth and the preservation of a sufficient sperm quality in order to find possible alternatives for conventional antibiotics used in the pig AI industry.

## Materials and methods

### Chemicals

Unless otherwise stated all chemicals used in this study were of analytical grade. Chemicals for spermatological analyses were purchased from Merck (Darmstadt, Germany) and Roth (Karlsruhe, Germany). Propidium iodide (PI) and Rhodamine 123 (R123) were obtained from Sigma-Aldrich (Steinheim, Germany), whereas fluorescein-isothiocyanate conjugated peanut agglutinin (FITC-PNA) and *Pisum sativum* agglutinin (FITC-PSA) were purchased from Axxora (Lörrach, Germany). All boar semen extenders were obtained from Minitüb (Tiefenbach, Germany).

### Synthesis and purification of lipopeptides

Seven short lipopeptides were assembled by solid-phase procedure using Fmoc chemistry on Fmoc-Rink Amide AM resin (0.48 mmol/g, IrisBiotech, Germany). Two amino acid derivatives, Fmoc-Lys(Boc)-OH and Fmoc-Lys(Fmoc)-OH (IrisBiotech, Germany), were used. The Fmoc group was detached by treating the resin with a solution of 20% piperidine in *N*, *N***-**dimethylformamide for 20 min (two steps of 5 and 15 min). Peptide bond formation was conducted with 1,2-diisopropylcarbodiimide (DIC)/1-hydroxybenzotriazole (HOBt) in dimethylformamide (DMF)/dichloromethane (DCM) (50/50; v/v) during 120 min. After each coupling and deprotection step, the peptidyl-resin was washed (3 × DCM, 3 × DMF/DCM, 3 × DCM). The deprotection and coupling reactions were monitored by the chloranil test. The final deprotection and cleavage of the lipopeptide from the solid support were performed with a solution of trifluoroacetic acid (TFA), triisopropylsilane (TIS) and water-TFA/TIS/water (90/2.5/2.5, v/v/v) for 120 min, at ambient temperature. After this, resin was filtered and the solution with cleaved lipopeptides was concentrated via rotary evaporator. The lipopeptides were then precipitated with diethyl ether and centrifuged. The precipitates were dissolved in water and freeze-dried (Christ, Germany). The lipopeptides were purified via reverse-phase high performance liquid chromatography (RP-HPLC) on a Knauer K1001 two-pump system (Knauer, Germany). For purification a semi-preparative column Nucleodur C18HTec (5 µm, 100 Å, 10 × 250 mm) was used (Macherey–Nagel, Germany). The lipopeptide compounds were eluted with a linear gradient with 20–60% of phase B (where phase A-0.1% TFA in water, phase B-0.1% TFA in acetonitrile), at a flow rate of 3 ml/min, at 214 nm for 120 min.

### Reversed-phase analysis of lipopeptides

The lipopeptides were analysed via RP-HPLC on a Chromolith Performance RP18ec (100 × 4.6 mm) column (Merck, Germany) using a Shimadzu Prominence system with a linear gradient with 2–98% phase B (where phase A-0.1% TFA in water, phase B-0.1% TFA in acetonitrile), at a flow rate of 2 ml/min, at 214 nm. Identity and purity of the lipopeptides were verified by the matrix assisted laser desorption ionization time-of-flight mass spectrometry (MALDI-TOF MS, Biflex III, Bruker, Germany). The purity of the lipopeptides was established higher than 95%.

### Experimental design

Two independent experiments (exp.) were conducted. In exp. 1, we evaluated potential detrimental effects of two concentrations of different lipopeptides on boar spermatozoa (Table 1). The minimum inhibitory concentration (1 × MIC) and 2 × MIC, which were established in prior studies on a range of different bacterial species ^17^, were used in antibiotic-free Beltsville Thawing Solution (BTS) extender (Table 2). Analyses of mitochondria activity (MITO), acrosome and plasma membrane integrity (PMAI) and progressive motility during thermo-resistance test (TRT) were done after 72 h of semen storage at 17 °C.

**Table 1:** Positive net charge, molecular weight (MW, calculated and determined by MS), hydrophilicity (clogP), critical micellar concentration (CMC) and minimum inhibitory concentration (MIC) of the investigated lipopeptides ^17^.

Lipopeptide	Positive net charge	MW [Da]_calc_	MW [Da]_MS_	clogP ALOGPs 2.1	CMC [mM]	MIC [µg/ml]
(C10)_2_-KKKK-NH_2_	3	837.5	838.5	3.30	1.76	16
C16-KKKK-NH_2_	4	767.6	768.6	2.64	14.60	8
C16-KKK-NH_2_	3	639.5	640.5	3.54	4.80	8
C16-KK-NH_2_	2	511.5	512.5	4.50	1.07	8
C14-KKKK-NH_2_	4	739.2	740.2	2.02	18.04	128
C14-KKK-NH_2_	3	611.5	612.5	2.84	11.70	64
C14-KK-NH_2_	2	483.3	484.3	3.75	4.40	64

**Table 2:** Description of the experimental groups in experiment 1 and 2.

Experiment 1	Extender	Temperature	Supplements (1 × MIC + 2 × MIC)
Control	BTS w/ gentamicin	17 °C 17 °C	–
Variants	BTS w/ gentamicin		(C10)2-KKKK-NH2 C16-KKKK-NH2 C16-KKK-NH2 C16-KK-NH2 C14-KKKK-NH2 C14-KKK-NH2 C14-KK-NH2
**Experiment 2**			
Negative control	BTS w/ gentamicin	17 °C	–
Positive control	ASP w/o antibiotics	17 °C	–
	ASP w/o antibiotics	5 °C	–
Variants	ASP w/o antibiotics	17 °C	C16-KKK-NH_2_ C16-KKKK-NH_2_
	ASP w/o antibiotics	5 °C	C16-KKK-NH_2_ C16-KKKK-NH_2_

In exp. 2, we examined the effects of 1 × MIC and 2 × MIC of two selected lipopeptides with the least negative impact on sperm quality, C16-KKK-NH_2_ and C16-KKKK-NH_2_, in extended boar semen stored at 17 °C and/or 5 °C (Table 2). In this trial, we used the antibiotic-free Androstar Premium (ASP) long-term extender (in treatment groups and positive control w/o supplements) and a conventional BTS extender with gentamicin sulphate (0.25 g/l, negative control, stored at 17 °C only). Analyses were the same as in exp. 1. Additionally, aliquots for microbiological investigations were taken immediately after sample preparation (0 h), and after 24 h, 48 h and 72 h of storage.

### Animals, semen collection and transport

All procedures involving animals were carried out in accordance with guidelines and regulations according to the European Commission Directive for Pig Welfare and were approved by the animal welfare committee of the state of Brandenburg (TVO-2019-V-20). A total of 14 ejaculates from different mature, healthy Pietrain boars (age: 16.9 ± 4.5 months) of proven fertility from a single boar stud in Northern Germany were used (exp. 1: n = 6 ejaculates; exp. 2: n = 8 ejaculates). The boars were routinely used for AI, received commercial feed (pellets) for AI boars and were housed individually in straw-bedded pens equipped with nipple drinkers. Ejaculates were collected weekly by the gloved-hand method. The day of collection is specified as day 0 (0 h of storage). The pre-sperm phase of each ejaculate was discarded and the gel fraction of the semen was removed by gauze filtration during collection.

Normospermic ejaculates were diluted isothermically (33 °C) on split-sample basis in order to prepare the following split semen sample groups (Table 2) according to extender type (BTS or ASP) and storage temperature (17 °C or 5 °C) using: BTS w/o antibiotics (exp. 1), BTS with gentamicin sulphate (0.25 g/L, control group in exp. 2) or Androstar Premium w/o antibiotics (2^nd^ control group and treatment group in exp. 2) to a concentration of 2.6 × 10^7^ spermatozoa/ml (NucleoCounter SP-100, Chemometec, Denmark). Semen was filled in QuickTip Flexitubes (Minitüb) with an AI dose volume of 85 ± 1 ml. Samples for 17 °C storage were placed in a temperature-controlled box at 22 °C for 90 min. The temperature was then reduced to 17 °C (overall cooling rate 4 °C/hour) and samples were stored in a refrigerator at 17 °C in the dark. Samples for 5 °C storage were placed in a cardboard box with a lid together with isothermic (28 °C) water-filled tubes. Boxes were subsequently kept for 6 h at 22 °C and then stored in a refrigerator at 5 °C in the dark according to Waberski et al.^15^.

### Sample preparation

The lipopeptides (Table 1) were prepared as 10 N solutions of 1 × MIC and 2 × MIC in semen extenders. One millilitre of lipopeptide solution was added to 10 ml of extended boar semen. Control samples did not contain further supplementation. For exp. 1, there was one sample per extract and concentration (control: BTS w/o gentamicin, samples: BTS w/o gentamicin w/ lipopeptides in 1 × and 2 × MIC, storage only at 17 °C).

Due to the experimental design (Table 2), exp. 2 required two samples of each extract-concentration combination (17 °C and 5 °C). In detail, exp. 2 had a negative control (BTS w/ gentamicin, 17 °C = BTS 17 °C), two positive controls (ASP w/o antibiotics, 17 °C and 5 °C = ASP 17 °C/ASP 5 °C) and supplemented samples (ASP w/o antibiotics + w/ lipopeptides in 1 × and 2 × MIC as well as 17 °C and 5 °C = 1 × /2 × MIC C16-KKK-NH_2_ / C16-KKKK-NH_2_ at 17 °C/5 °C in all six combinations).

### Evaluation of boar sperm motility

Motility assessment was done using a computer-assisted semen analysis (CASA, AndroVision, Minitüb). Samples for thermo-resistance test were measured after 72 h of semen storage and a following incubation time of 30 min (TRT30) and 300 min (TRT300) at 38 °C according to Schulze et al*.*^19^. Subsamples of 3.0 µl each were placed in preheated Leja chambers (Leja Products B.V., Nieuw Vennep, The Netherlands) and at least 1,000 spermatozoa per sample were recorded. Spermatozoa were defined motile when showing an amplitude of lateral head displacement (ALH) > 1.0 µm and a velocity curved line (VCL) > 24.0 µm x s^-1^. When VCL was ≥ 48.0 µm x s^-1^ and velocity straight line (VSL) was ≥ 10.0 µm x s^-1^, they were listed as progressively motile spermatozoa.

### Flow cytometric assessment of boar spermatozoa

All aliquots for analyses were taken after 72 h of storage and 30 min of incubation at 38 °C. Analyses were performed using CytoFLEX S (Beckman Coulter, Indianapolis, United States) equipped with a 488 nm and 525 nm diode laser. Fluorescence signals of FITC-PNA, FITC-PSA and R123, gathered via 525/40 nm BP filter, and PI, gathered via 610/20 nm BP filter, were plotted on logarithmic scales. The sperm population was gated referring to the expected forward- and side-scatter signals and a total of 10,000 events were counted in this area. For assessment of MITO, a double-staining with R123 and PI according to Schulze et al.^20^ was done. To evaluate PMAI, a triple-staining with FITC-PNA, FITC-PSA and PI was done as described previously^21^. After incubation with fluorochromes, 30 µl of each sample were added to 2 ml of isotherm phosphate-buffered NaCl solution. The percentage of spermatozoa with active mitochondria (MITO) and the percentage of spermatozoa with intact acrosome and plasma membrane (PMAI) were recorded.

### Microbiological analyses

In exp. 2, immediately after sampling (0 h) as well as after 24 h, 48 h and 72 h of semen storage, two aliquots of 1.5 ml per sample were taken from samples with lipopeptide supplementation in 1 × MIC as well as from 17 °C BTS w/ gentamicin and 5 °C ASP w/o supplements. Aliquots of extended semen were frozen with glycerol 5:1 (vol/vol) in liquid nitrogen before storage at -80 °C until analysis to protect bacterial cells from freeze–thaw damage^22^. To determine the total microbial count in extended semen samples, serial dilutions were prepared in PBS ranging from 10^–1^ to 10^–2^. 100 µl of each dilution were plated on LB agar plates (LB medium from Carl Roth) in duplicates and incubated at 37 °C under aerobic conditions. Colony forming units (CFU)/ml were calculated after 48 h of incubation.

Bacterial isolations were performed from 10 µl of extended semen samples on a plate set routinely used for bacterial diagnostics (Columbia Agar with sheep blood, Gassner and UTI Clarity agar, all Oxoid Thermo Fisher, Wesel, Germany). The agar plates were incubated aerobically for 24 h at 37 °C and the initial blood agar plates were re-incubated for another 24 h at 37 °C under 5% CO_2_ atmosphere to promote slow and fastidious growing bacterial species. After 24 h and 48 h incubation, distinct bacterial colonies were subcultured under identical conditions for bacterial identifications. Bacterial isolates were identified by standard diagnostic methods including the API identification system (BioMerieux, Nürtingen, Germany) and 16S rDNA gene analysis according to Mühldorfer et al*.* (2011)^23^. All subcultures were finally grouped into three major categories: (i) Gram-positive rods, (ii) Gram-positive cocci and (iii) Gram-negative species for comparison of the results. The grouping was necessary because of the diversity of the isolated bacterial species and several isolates that could not be fully classified to genus or species levels.

### Statistical analysis

**Figure 1:**
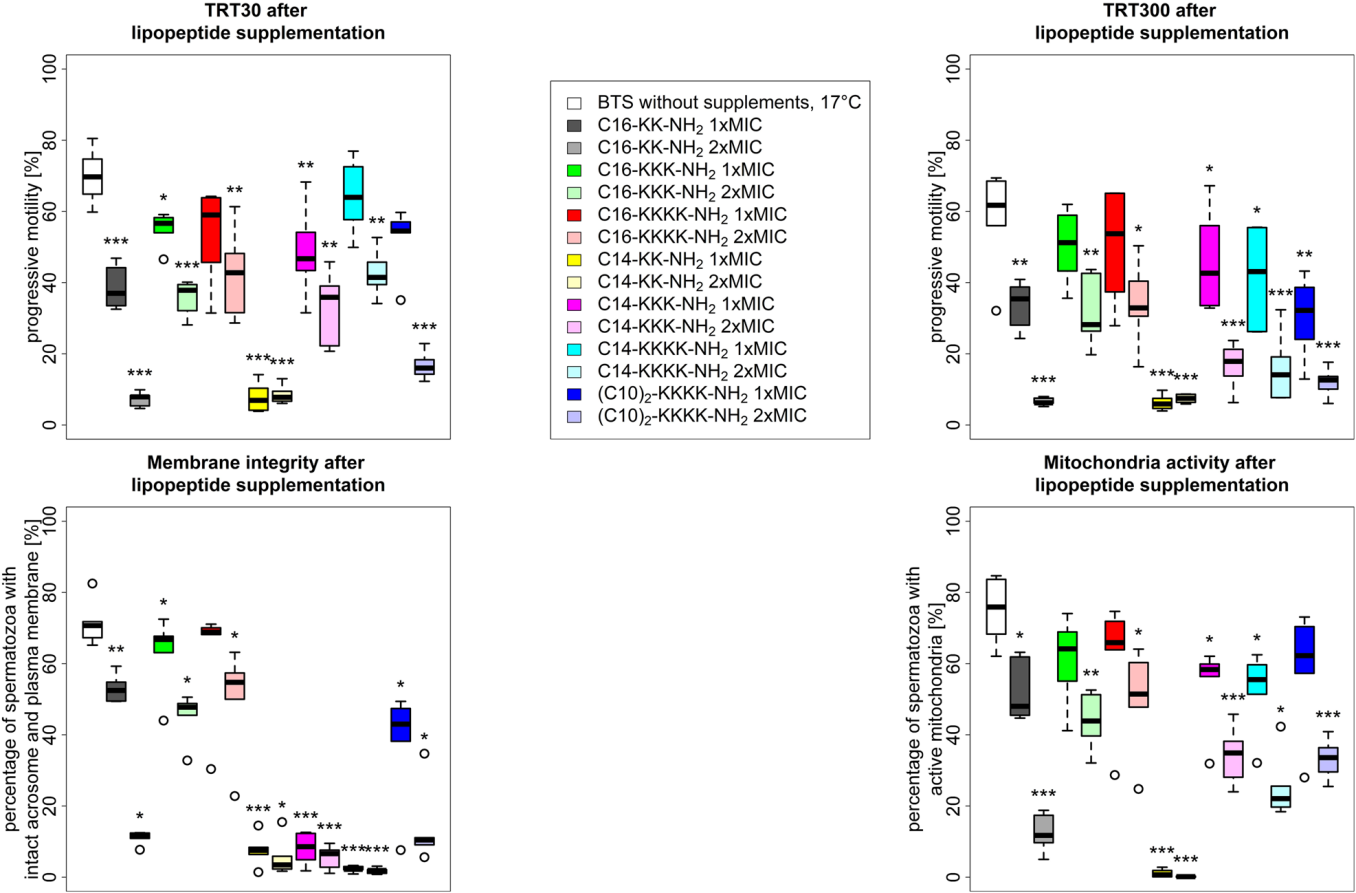
Sperm quality characteristics (TRT30, TRT300, MITO, PMAI) after 72 h storage at 17 °C in antibiotic-free BTS (= control) with lipopeptide supplementation (n = 6 ejaculates). The following seven lipopeptides were supplemented at two concentrations each (1 × MIC / 2 × MIC): C16-KK-NH_2_, C16-KKK-NH_2_, C16-KKKK-NH_2_, C14-KK-NH_2_, C14-KKK-NH_2_, C14-KKKK-NH_2_ and (C10)_2_-KKKK-NH_2_. Stars above the boxplots indicate significant differences to BTS (*P < 0.05, **P < 0.01; ***P < 0.001). MIC = minimum inhibitory concentration, TRT = thermo-resistance test.

**Figure 2:**
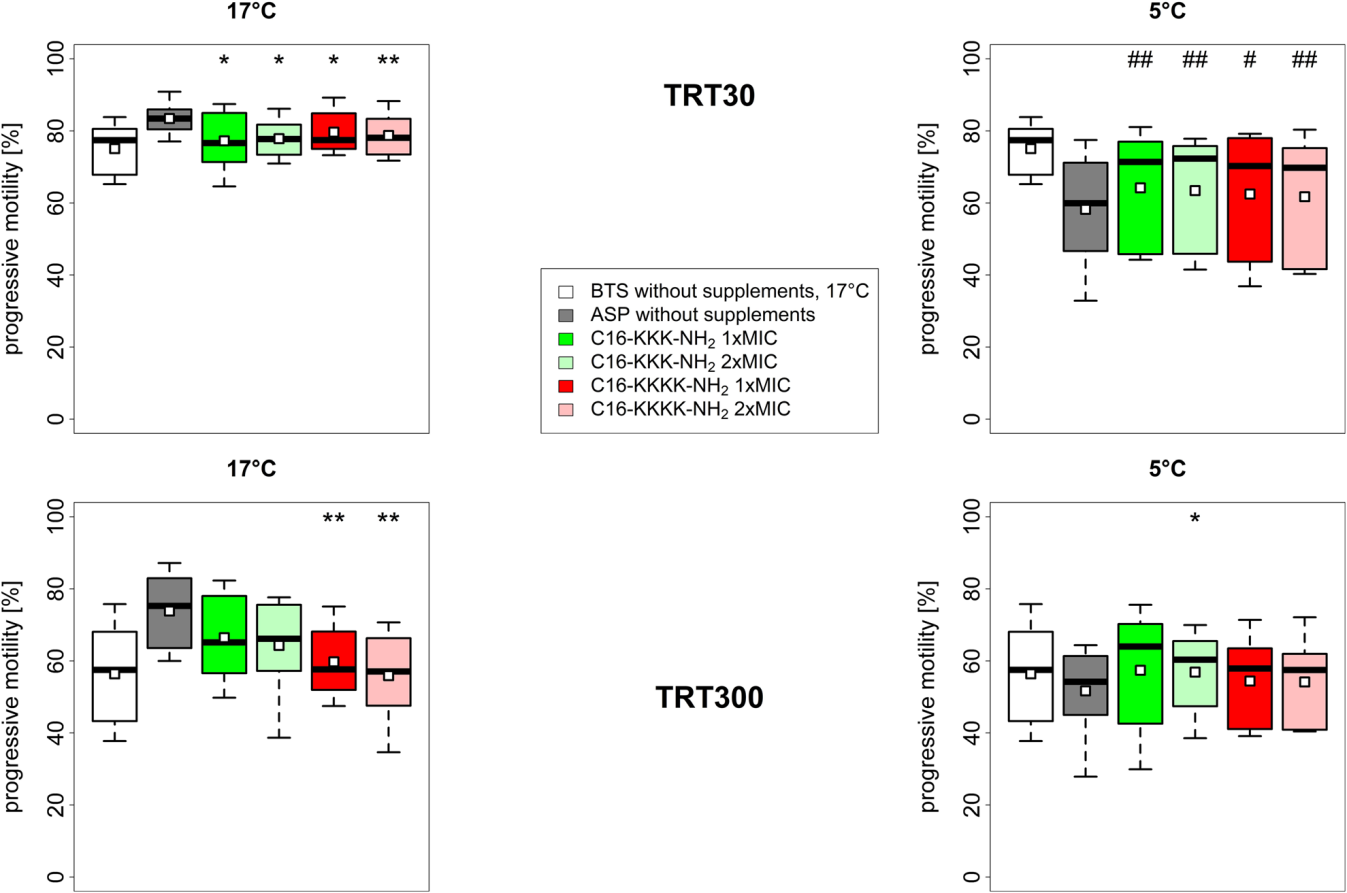
Sperm quality characteristics (TRT30, TRT300) after 72 h storage at 17 °C or 5 °C (n = 8 ejaculates). Sperm were stored in BTS w/ gentamicin (= negative control, only 17 °C) or in ASP w/o supplements (= positive control) or in ASP w/ lipopeptide supplementation in 1 × MIC or 2 × MIC (C16-KKK-NH2 / C16-KKKK-NH2). Stars above the boxplots indicate significant differences to ASP in the corresponding temperature level (*P < 0.05, **P < 0.01). Hashtags above the boxplots indicate significant differences to BTS 17 °C (#*P* < 0.05, ##*P* < 0.01). Means are denoted as open squares. For better comparison, the BTS 17 °C control is shown in the 17 °C graphs as well as in the 5 °C graphs. MIC = minimum inhibitory concentration, TRT = thermo-resistance test.

**Figure 3:**
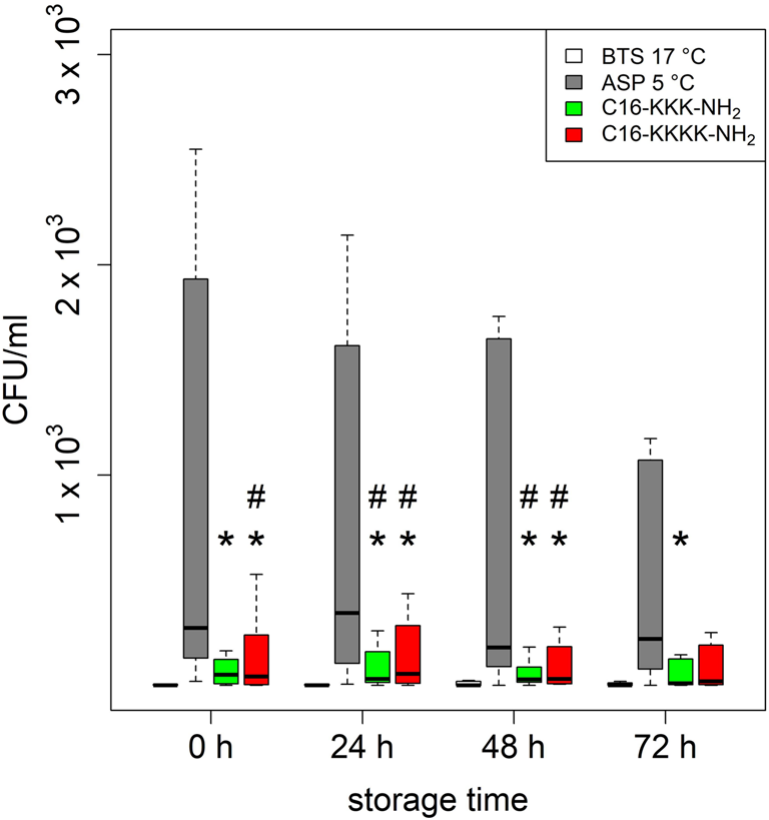
Effect of storage time on the bacterial load in liquid preserved boar semen (n = 8 ejaculates). Colony forming units (CFU)/ml were analysed after 0 h, 24 h, 48 h and 72 h of storage either at 17 °C in BTS w/ gentamicin (negative control) or at 5 °C in ASP w/o supplements (positive control) or in ASP w/ lipopeptide supplementation in 1 × MIC of C16-KKK-NH2 or C16-KKKK-NH2. Stars above the boxplots indicate significant differences to ASP (*P < 0.05). Hashtags above the boxplots indicate significant differences to BTS 17 °C (#*P* < 0.05). For better visibility, the outliers were excluded from the figure.

Data analysis was performed using SPSS Statistics 23 (IBM, Armonk, USA) and R (R Foundation for Statistical Computing, Vienna, Austria)^24^. Sperm quality characteristics in exp. 1 and 2 as well as the microbiological data were directly compared between each supplement and control. Data were tested on normal distribution (Shapiro–Wilk test) and variance homogeneity (Levene test) before choosing the appropriate paired test (normal, variance homogeneous: t-test, not homogeneous: Welch test, non-normal: Mann–Whitney-U test). The resulting significances are depicted in Figs. 1–3. To meet the requirements of repeated measures experimental design of exp. 2, a generalized linear mixed model was applied. Therefore, the AI boars were appointed as experimental samples (subjects) and the extender (BTS w/ gentamicin, ASP w/o supplements, ASP w/ C16-KKK-NH_2_, ASP w/ C16-KKKK-NH_2_), the concentration (1 × MIC, 2 × MIC), the storage temperature (17 °C, 5 °C) and the incubation time (30 min, 300 min) were considered as fixed factors. Data are presented as boxplots. Differences were considered significant when the calculated probability of their occurrence was less than 5% (*P* < 0.05).

## Results

### Experiment 1—screening of lipopeptides

The results of exp. 1 are shown in Fig. 1. Five of seven lipopeptides (C16-KK-NH_2_, C14-KK-NH_2_, C14-KKK-NH_2_, C14-KKKK-NH_2_, (C10)_2_-KKKK-NH_2_) had negative effects on most of the tested sperm quality characteristics in comparison to the control (BTS w/o antibiotics 17 °C). Particularly, 2 × MIC variants appeared to have detrimental effects on sperm quality. The lipopeptides C16-KK-NH_2_, C14-KK-NH_2_ and C14-KKK-NH_2_ had deteriorating effects on all evaluated sperm quality characteristics in both 1 × MIC (MITO: *P* ≤ 0.031; PMAI: *P* ≤ 0.004; TRT30: *P* < 0.001; TRT300: *P* ≤ 0.005) and 2 × MIC (PMAI: *P* = 0.031; others: *P* < 0.001). There was a similar trend for the lipopeptides C14-KKKK-NH_2_ and (C10)_2_-KKKK-NH_2_ on all analysed sperm characteristics in 2 × MIC, whereas in 1 × MIC one or two sperm quality characteristics were unaffected. This became apparent in the following cases: C14-KKKK-NH_2_ had no effect on TRT30 and (C10)_2_-KKKK-NH_2_ did not reduce MITO or TRT30. While the other two lipopeptides, C16-KKK-NH_2_ and C16-KKKK-NH_2_, also caused sperm quality impairment on the analysed characteristics in 2 × MIC (MITO: *P* ≤ 0.021; PMAI: *P* ≤ 0.031; TRT30: *P* ≤ 0.005; TRT300: *P* ≤ 0.020), their effect on sperm quality in 1 × MIC was minor. Here, only C16-KKK-NH_2_ affected PMAI (*P* = 0.031) and TRT30 (*P* = 0.027), whereas C16-KKKK-NH_2_ did not negatively impair the tested sperm quality characteristics at all.

### Experiment 2

#### Spermatological results

In exp. 1, the two lipopeptides C16-KKK-NH_2_ and C16-KKKK-NH_2_ qualified for further investigations based on their overall low cytotoxicity. Altogether, the applied extender (w/ and w/o supplements), the storage temperature, the lipopeptide concentration and the incubation time had significant effects on sperm quality (Tables 3, 4). The comparison between BTS w/ gentamicin (BTS 17 °C) and ASP w/o supplements (ASP) showed a significant effect in favour of ASP regarding the progressive motility at 17 °C (Table 3, *P* = 0.001). The two tested lipopeptides C16-KKK-NH_2_ and C16-KKKK-NH_2_ did neither negatively affect progressive motility nor MITO or PMAI compared to BTS 17 °C (Table 3). Comparing ASP w/o supplements and ASP w/ lipopeptides, both AMPs significantly reduced progressively motile spermatozoa (Table 4, *P* = 0.019 and *P* = 0.012, respectively) and PMAI (both *P* = 0.001). Furthermore, C16-KKK-NH_2_ reduced MITO when compared to ASP w/o supplements (Table 4, *P* = 0.021). The concentration of lipopeptides had a significant effect on PMAI. Here, it became apparent that 1 × MIC resulted in a higher percentage of membrane intact spermatozoa (Table 3, *P* = 0.006) compared to 2 × MIC. Furthermore, the temperature had a significant effect with a reduction of the overall progressive motility, MITO and PMAI after storage at 5 °C (Table 3, each *P* < 0.001).

**Table 3:** Results of a generalized linear mixed model for the effect of different factors on progressive motility in a thermo-resistance test, mitochondria activity and membrane integrity of boar spermatozoa preserved for 72 h when compared to the negative control (BTS w/ gentamicin, 17 °C).

Sperm characteristic	Model term	Coefficient	Sig	95% confidence interval	
				Lower	Upper
Progressive motility	Intercept	60.67	0	55.47	65.86
	Extender (+ supplement)		**0.004**		
	BTS w/ gentamicin	0			
	ASP w/o supplements	8.95	0.001	3.74	14.16
	ASP w/ C16-KKK-NH_2_	4.35	0.107	− 0.95	9.65
	ASP w/ C16-KKKK-NH_2_	3.83	0.163	− 1.57	9.23
	Concentration			**0.785**	
	0 × MIC	0			
	1 × MIC	0.48	0.785	− 2.96	3.91
	2 × MIC	0			
	Temperature			** < 0.001**	
	5 °C	− 13.32	< 0.001	− 17.17	− 9.47
	17 °C	0			
	Incubation time			** < 0.001**	
	30 min	13.37	< 0.001	10.04	16.70
	300 min	0			
Mitochondria activity	Intercept	82.36	0	78.09	86.63
	Extender (+ supplement)		**0.456**		
	BTS w/ gentamicin	0			
	ASP w/o supplements	2.59	0.313	− 2.48	7.66
	ASP w/ C16-KKK-NH_2_	− 1.81	0.472	− 6.78	3.17
	ASP w/ C16-KKKK-NH_2_	− 0.80	0.748	− 5.73	4.13
	Concentration			**0.250**	
	0 × MIC	0			
	1 × MIC	1.58	0.250	− 1.14	4.30
	2 × MIC	0			
	Temperature			** < 0.001**	
	5 °C	− 9.25	< 0.001	− 13.77	− 4.72
	17 °C	0			
Membrane integrity	Intercept	82.93	0	79.90	85.95
	Extender (+ supplement)		**0.184**		
	BTS w/ gentamicin	0			
	ASP w/o supplements	2.58	0.157	− 1.01	6.18
	ASP w/ C16-KKK-NH_2_	− 3.91	0.084	− 8.37	0.54
	ASP w/ C16-KKKK-NH_2_	− 2.30	0.219	− 5.98	1.39
	Concentration			**0.006**	
	0 × MIC	0			
	1 × MIC	3.95	0.006	1.14	6.76
	2 × MIC	0			
	Temperature			** < 0.001**	
	5 °C	− 14.20	< 0.001	− 18.71	− 9.70
	17 °C	0			

The model included the extender (ASP w/o supplements) and lipopeptides (C16-KKK-NH_2_ or C16-KKKK-NH_2_), the concentration (0 × MIC / 1 × MIC / 2 × MIC), the temperature (5 °C / 17 °C) and the incubation time (30 min / 300 min) as fixed effects. Results are shown as model coefficient (with 95% confidence interval) with significance (Sig.) of the general factor (bold) and the specific factor levels (non-bold).

**Table 4:** Results of a generalized linear mixed model for the effect of different factors on progressive motility in a thermo-resistance test, mitochondria activity and membrane integrity of boar spermatozoa preserved for 72 h when compared to the positive control (ASP w/o supplements).

Sperm characteristic	Model term	Coefficient	Sig	95% confidence interval	
				Lower	Upper
Progressive motility	Intercept	69.61	0	65.72	73.51
	Extender (+ supplement)		**0.004**		
	BTS w/ gentamicin	− 8.95	0.001	− 14.16	− 3.74
	ASP w/o supplements	0			
	ASP w/ C16-KKK-NH_2_	− 4.60	0.019	− 8.43	− 0.77
	ASP w/ C16-KKKK-NH_2_	− 5.12	0.012	− 9.10	− 1.15
Mitochondria activity	Intercept	84.95	0	82.21	87.69
	Extender (+ supplement)		**0.456**		
	BTS w/ gentamicin	− 2.59	0.313	− 7.66	2.48
	ASP w/o supplements	0			
	ASP w/ C16-KKK-NH_2_	− 4.39	0.021	− 8.10	− 0.69
	ASP w/ C16-KKKK-NH_2_	− 3.39	0.069	− 7.04	0.27
Membrane integrity	Intercept	85.51	0	83.56	87.46
	Extender (+ supplement)		**0.184**		
	BTS w/ gentamicin	− 2.58	0.157	− 6.18	1.01
	ASP w/o supplements	0			
	ASP w/ C16-KKK-NH_2_	− 6.49	0.001	− 10.27	− 2.72
	ASP w/ C16-KKKK-NH_2_	− 4.88	0.001	− 7.72	− 2.03

The model included the extender (BTS w/ gentamicin) and lipopeptides (C16-KKK-NH_2_ or C16-KKKK-NH_2_), the concentration (0 × MIC / 1 × MIC / 2 × MIC), the temperature (5 °C / 17 °C) and the incubation time (30 min / 300 min) as fixed effects. Results are shown as model coefficient (with 95% confidence interval) with significance (Sig.) of the general factor (bold) and the specific factor levels (non-bold).

Based on the differences from the mixed model, we performed a paired test between each variant and control shown in Fig. 2. In a direct comparison between lipopeptide supplemented samples and 17 °C BTS w/ gentamicin (negative control), the progressive motility in TRT30/300 was not significantly different after 17 °C semen storage, but TRT30 was significantly lower after 5 °C semen storage for both lipopeptide variants (C16-KKK-NH_2_: *P* = 0.008, C16-KKKK-NH_2_: *P* ≤ 0.023). The effect was no longer observed after TRT300. Compared to ASP w/o supplements (positive control) at 17 °C semen storage, both lipopeptide variants showed a reduced motility after TRT (TRT30: C16-KKK-NH_2_: *P* = 0.014 and C16-KKKK-NH_2_: *P* ≤ 0.012; TRT300: C16-KKK-NH_2_: non-significant and C16-KKKK-NH_2_: *P* ≤ 0.005). After TRT30/300 at 5 °C semen storage, progressive motility was not reduced and even slightly enhanced, especially in TRT300 with 2 × MIC C16-KKK-NH_2_ (*P* = 0.012).

#### Microbiological results

Wide, boar-individual ranges of the total bacterial load were determined from the investigated samples (Fig. 3). The number of bacteria in samples stored in BTS w/ gentamicin at 17 °C (negative control) was significantly different (0 & 24 h: *P* = 0.008 and 48 & 72 h: *P* = 0.022) compared to ASP w/o supplements at 5 °C (positive control). ASP w/ lipopeptide variants showed a strongly reduced bacterial load compared to the positive control, the results were significant at almost all storage times (C16-KKK-NH_2_: 0 h: *P* = 0.023, 24 h: *P* = 0.035, 48 h: *P* = 0.023, 72 h: *P* = 0.023; C16-KKKK-NH_2_: 0 h: *P* = 0.023, 24 h: *P* = 0.039, 48 h: *P* = 0.023, 72 h: *P* = 0.055).

The composition of the bacterial profile is shown in Table 5. The ASP extender w/o supplements at 5 °C (positive control) showed the highest number of distinct subcultures identified over all time points, with a total of 103 and a mean of 13, whereas the supplementation with C16-KKK-NH_2_ and C16-KKKK-NH_2_ resulted in a total of 56 and 67 distinct subcultures and a mean of 7 and 8, respectively. A reduction of the number of subcultures due to the lipopeptides was noticeable in all categories of bacteria, with a decrement from 65 to 39/52 total subcultures of Gram-positive rods, from 21 to 9/10 total subcultures of Gram-positive cocci and from 17 to 8/5 total subcultures of Gram-negative species, respectively.

**Table 5:** Bacterial spectrum after 72 h storage of boar spermatozoa (n = 8 ejaculates) at 5 °C in ASP w/o supplements (positive control) or in ASP w/ supplemented with 1 × MIC C16-KKK-NH_2_ and C16-KKKK-NH_2_ (exp. 2).

	ASP w/o supplements			ASP w/ C16-KKK-NH_2_			ASP w/ C16-KKKK-NH_2_		
	Total numbers	mean	% of total subcultures	Total numbers	mean	% of total subcultures	Total numbers	mean	% of total subcultures
Total subcultures	103	13	100.00	56	7	100.00	67	8	100.00
Gram-positive rods	65	8	63.11	39	5	69.64	52	7	77.61
Gram-positive cocci	21	3	20.39	9	1	16.07	10	1	14.93
*Staphylococcus* spp.	5	1	4.85	3	0	5.36	2	0	2.99
*Streptococcus* spp.	4	1	3.88	4	1	7.14	5	1	7.46
Gram-negative species	17	2	16.50	8	1	14.29	5	1	7.46
Non-fermenters	11	1	10.68	8	1	14.29	3	0	4.48
Fermenters	6	1	5.83	0	0	0.00	2	0	2.99

Additionally, bacterial species of potential concern for AI were recorded (Table 6). This category included species that could negatively impair the spermatozoa or bear specific risks in semen production^25^, such as potential extended spectrum beta-lactamase (ESBL) or biofilm producing bacteria, as well as species that can cause bacterial infections in sows. The following species from this category were identified in ASP-extended semen samples: *Trueperella pyogenes*, *Streptococcus (S.) porcinus*, *Alcaligenes faecalis*, *Pseudomonas* (*P.*) *aeruginosa, Pasteurella* sp., *Providencia stuartii* and *Escherichia* (*E.*) *coli*. They were also isolated from the positive control (ASP 5 °C w/o lipopeptides) with the exception of *E. coli*. In samples supplemented with C16-KKKK-NH_2_, only *S. porcinus* and *E. coli* were detected, and in samples containing C16-KKK-NH_2_ only *P. aeruginosa* was found.

**Table 6:** Bacterial species of potential concern in artificial insemination identified from ASP samples w/o supplementation (positive control) and from ASP samples with lipopeptide supplementation (C16-KKK-NH_2_ or C16-KKKK-NH_2_ in 1 × MIC) after storage at 5 °C for 72 h.

Bacteria	ASP w/o supplements	ASP w/ C16-KKK-NH_2_	ASP w/ C16-KKKK-NH_2_
*Trueperella pyogenes*	1		
*Streptococcus porcinus*	1		1
*Alcaligenes faecalis*	1		
*Pseudomonas aeruginosa*	3	1	
*Pasteurella* sp.	2		
*Providencia stuartii*	2		
*Escherichia coli*			2
Sum of bacterial species	10	1	3
Total subcultures	103	56	67
% of bacteria on total subcultures	10	2	4

The frequency of each species as well as the sum and the percentage in proportion to the total subcultures are shown.

## Discussion

Currently, the pig AI industry is heavily relying on the routine use of antibiotics in semen preservation in order to maintain high standards of sperm quality from production to insemination. The steadily increasing threat of antimicrobial resistance, however, is an urgent matter that calls for plans of action in all related areas of concern. The possibility of a boar semen preservation procedure that could forgo the use of antibiotics while still maintaining satisfactory sperm quality would therefore be an important step in combatting multidrug-resistant bacteria. The aim of our study was to investigate, whether the combination of a low temperature storage at 5 °C with the use of lipopeptides as alternative antimicrobial supplements could meet these conditions.

The results of exp. 1 revealed that five out of seven lipopeptides impaired more than one of the tested sperm characteristics in a significant matter and were therefore excluded from the follow-up experiment. The effects were concentration-dependent, as 2 × MIC had more severe negative effects than 1 × MIC on sperm quality. This observation is in accordance with other studies implying that the concentration in which the AMPs are applied is an important determining factor for the sperm compatibility of antimicrobial agents^26^. A probable reason for the interference of five of the tested lipopeptides with sperm quality is their antimicrobial mode of action, which not only targets unwanted bacteria, but also negatively impacts sperm physiology and therefore prohibits their use in semen preservation.

There are different assumptions as to how AMPs operate on a molecular level. Regarding their primary mode of action, AMPs can be classified as either membrane targeting or non-membrane targeting^27,28^. In most cases, membrane targeting ultimately leads to an increase in membrane permeability and thus deregulation of the membrane potential^29,30^. The observations from exp. 1 show that the membrane integrity of spermatozoa was severely affected by almost all tested lipopeptides, suggesting that the cytotoxic effects on the spermatozoa are based on membrane-targeting peptide properties. It is very likely that the same mechanism can be assumed for their interaction with bacteria. In general, the two most important factors for the membrane selectivity of AMPs towards prokaryotic cells are the electrostatic interaction between the cationic peptide and the highly acidic bacterial membrane, and secondly the presence of a relatively high amount of membrane-stabilizing cholesterol in eukaryotic cells, causing a higher rigidity of the lipid bilayer and thus inhibiting membrane disruption through AMPs^12^. However, due to their relatively low ratio of membrane cholesterol to membrane phospholipids and the exposure of anionic sulfogalactosylglycerolipid on the sperm surface, boar sperm cells are more likely to be targeted by membrane-active AMPs than most other eukaryotic cells^14^. Again, this became apparent when five out of the seven tested lipopeptides caused sperm quality impairment to a non-acceptable degree.

Two lipopeptides (C16-KKK-NH_2_ and C16-KKKK-NH_2_) from this study impaired the sperm quality less severely than the other five. Interestingly, both belonged to the group of lipopeptides with the lowest MIC against tested control bacteria^18,31^, suggesting, that the reason for their sperm-compatibility was not an overall lower efficiency or activity, but rather a higher and more specific target selectivity. While there is evidence in prior studies suggesting that the effect of the two selected lipopeptides results from a more complex mode of action than a simple detergent-like membrane disruption, their intricate mechanism is yet to be discovered^32^. Nevertheless, AMPs bear a high potential to be applied as additives in semen preservation, when carefully selected on the basis of their antimicrobial mechanism of action and membrane selectivity.

A second important approach for antibiotic-free boar semen storage is the possibility of a low temperature preservation, which has been studied extensively but with limited success due to the high cold shock susceptibility of boar semen^4,33^. The most important mechanism for sperm quality loss due to lower storage temperatures is the lipid phase transition of the cell membrane^34^. Boar sperm are more prone to cold shock than sperm of many other species because of their relatively low concentration of membrane stabilizing cholesterol. However, the susceptibility for cold shock is dependent on many different factors, and recently there have been reports of successfully sustained sperm quality at low temperature through optimization of surrounding conditions^15,35,36^. Amongst others, boar individual eligibility^37,38^, the cooling rate^15,39^, the used extender^40^, motionless storage^41^ and transport^42^ play important roles in the maintenance of sperm quality.

Androstar Premium extender was used just as in other studies, where boar sperm were successfully stored at 5 °C^15,35^. Even though the precise composition of this extender is not revealed by the manufacturers, it is anticipated that membrane stabilizers and capacitation inhibitors help to preserve sperm quality for longer periods of time or in less favourable surrounding conditions. Our results support these propositions as samples stored in ASP showed significantly better sperm characteristics compared to BTS.

When considering the influence of the temperature, calculated with the linear mixed model, a “chilling-induced” negative effect on sperm cells became apparent. However, in a direct comparison of each 5 °C lipopeptide variant to the 17 °C BTS control w/ gentamicin (AI standard), no significant differences in progressive sperm motility could be found after thermo-resistance test, suggesting that surrounding conditions in the extender play an important role in the feasibility of a low temperature storage for commercial use. This observation can be considered a very positive effect, as the thermo-resistance is a sensitive fertility predictor, reflecting the relatively long period of exposure to 38 °C that spermatozoa face in the female reproduction tract before fertilization^20^. Furthermore, it is worth mentioning, that the mean progressive motility of all samples stored at 5 °C after 30 min incubation at 38 °C met the standard cut-off criteria (> 65% for sperm motility after 3 days of storage) for AI in Germany^43^. One could therefore conclude that, though accompanied by a slight impairment of certain quality characteristics, low temperature storage is still a feasible possibility, as the overall sperm quality is preserved to a satisfactory degree.

Another important factor that needs to be considered aside from the sperm quality is the effect of the chosen semen storage procedure on the bacterial load. It can be assumed that each AI dose has a basic bacterial content, since it is almost impossible to obtain a germ-free ejaculate. On average, boar ejaculates are loaded with 10^3^–10^5^ CFU/ml of bacteria^25^, but bacterial numbers are highly influenced by the experimental conditions such as study design, the breed and age of the boar, as well as the method of ejaculate collection^44,45^. The causes for initial bacterial contamination can be animal- and / or environment-associated^46,47^. Therefore, the goal is not necessarily the production of sterile AI doses but rather adequate hygiene measures and the reduction of bacterial growth during storage through effective antibacterial means. Standard storing procedures reach this goal by the supplementation of antibiotics according to legal guidelines (Council Directive, European Union, 90/429/EEC).

Even though the use of BTS extender w/ gentamicin resulted in the lowest amount of CFU/ml, the number of bacteria in samples supplemented with one of the two selected lipopeptides was below the critical value of 10^3^ CFU/ml^3,43^ at all tested time points. Furthermore, both lipopeptides and particularly the C16-KKKK-NH_2_ lipopeptide showed a selective effect on Gram-negative bacterial species, including ones often found in pig AI centers^2,25^. Additionally, the analysis regarding selected common species of concern in AI also showed that both lipopeptides clearly reduced the number of relevant Gram-negative and Gram-positive bacterial species from six (ASP w/o) to only two (C16-KKKK-NH_2_) or one (C16-KKK-NH_2_) compared to the controls, which are promising results for their antibacterial activity. Prior studies with similar lipopeptides support these findings, demonstrating that lipopeptides inhibit the growth of both Gram-positive and Gram-negative bacteria^48^ and the overall antimicrobial activity appeared to be dependent on the length of the hydrophobic chains and the amount of lysine residues^32^.

In conclusion, an antibiotic-free low temperature preservation of boar semen at 5 °C supplemented with one of the two selected lipopeptides would be possible with only minor impairments when compared to the standard preservation at 17 °C in BTS containing antibiotics. These limitations include a slightly impeded sperm quality due to the low temperature storage and a slightly higher bacterial load. Nevertheless, the results were still within the quality requirements for AI doses worldwide^43^, and the abstain from the use of antibiotics should be considered the most compelling argument in comparison to these minor quality impairments.
